# Neuropsychological functions and visual contrast sensitivity in schizophrenia: the potential impact of comorbid posttraumatic stress disorder (PTSD)

**DOI:** 10.3389/fpsyg.2013.00136

**Published:** 2013-03-20

**Authors:** Ibolya Halász, Einat Levy-Gigi, Oguz Kelemen, György Benedek, Szabolcs Kéri

**Affiliations:** ^1^National Psychiatry CenterBudapest, Hungary; ^2^Institute for the Study of Affective Neuroscience, University of HaifaHaifa, Israel; ^3^Psychiatry Center, Bács-Kiskun County HospitalKecskemét, Hungary; ^4^Department of Physiology, Faculty of Medicine, University of SzegedSzeged, Hungary

**Keywords:** schizophrenia, posttraumatic stress disorder (PTSD), neuropsychology neuroimaging, vision tests, psychopathological features

## Abstract

Previous studies have revealed a high prevalence of posttraumatic stress disorder (PTSD) in patients with other severe mental disorders, including schizophrenia. However, the neuropsychological and psychophysical correlates of comorbid PTSD are less exactly defined. The purpose of the present study was to assess immediate and delayed memory, attention, visuospatial skills, language, and basic visual information processing in patients with schizophrenia with or without PTSD. We recruited 125 patients with schizophrenia and 70 healthy controls matched for visual acuity, age, gender, education, and socioeconomic status. Twenty-one of patients with schizophrenia exhibited comorbid PTSD. We administered the Repeatable Battery for the Assessment of Neuropsychological Status (RBANS) and visual contrast sensitivity tasks for low spatial/high temporal frequency (0.3 cycle/degree and 18 Hz) and high spatial/low temporal frequency (10 cycles/degree and 1Hz) sinusoidal gratings. All patients were clinically stable and received antipsychotic medications. Results revealed that relative to healthy controls, patients with schizophrenia exhibited significant and generalized neuropsychological dysfunctions and reduced visual contrast sensitivity, which was more pronounced at low spatial/high temporal frequency. When we compared schizophrenia patients with and without PTSD, we found that patients with comorbid PTSD displayed lower scores for RBANS attention, immediate and delayed memory, and visuospatial scores. Schizophrenia patients with or without PTSD displayed similar visual contrast sensitivity. In conclusion, comorbid PTSD in schizophrenia may be associated with worse neuropsychological functions, whereas it does not affect basic visual information processing.

## Introduction

The presence, origin, and clinical characteristics of posttraumatic stress disorder (PTSD) in severe mental disorders, such as schizophrenia, are one of the most controversial issues in the literature. According to the meta-analysis of Achim et al. (2011), the prevalence of PTSD in schizophrenia and related psychotic disorders is 12.4%, with a wide confidence interval of 4–20.8%. Comorbid PTSD, which is characterized by upsetting re-experiencing the traumatic event (e.g., flashbacks and nightmares), avoidance of thoughts and cues related to the trauma, emotional numbing, and increased arousal, may be associated with adverse functioning, lower quality of life, and worse outcome (Grubaugh et al., 2011). Early trauma may be a pathogenic factor in the development of both schizophrenia and PTSD, but later traumatic events after the emergence of schizophrenia may also lead to PTSD (Schäfer and Fisher, 2011; Matheson et al., 2013).

In this study, we aimed to clarify two aspects of schizophrenia in relation to comorbid PTSD: early stage visual information processing and neuropsychological functions. There is a great deal of evidence that basic low-level vision, such as the detection of simple luminance-contrast gratings and elementary perceptual organization, is impaired in schizophrenia (reviewed by Javitt, 2009; Silverstein and Keane, 2011; Butler et al., 2012). Neuropsychological and social cognitive deficits are extensively documented in schizophrenia, exhibiting a definitive relationship with psychosocial functioning (Green, 2007; Fett et al., 2011; Keefe and Harvey, 2012). To our knowledge, there have been no studies investigating visual information processing in schizophrenia with comorbid PTSD, and reports on neuropsychological functions provided inconsistent results because of differences in methods and patient populations (Goodman et al., 2007; Fan et al., 2008; Duke et al., 2010; Peleikis et al., 2012). Therefore, we recruited a large sample of patients with schizophrenia and screened them for PTSD. We measured visual contrast sensitivity to characterize low-level perceptual functions and administered a standard battery of neuropsychological tests assessing immediate and delayed memory, attention, language, and visuospatial functions. We hypothesized that patients with schizophrenia with comorbid PTSD (SCZ + PTSD) would display worse visual and neuropsychological functions compared with schizophrenia patients without PTSD (SCZ). The hypothesis of early stage visual dysfunction was based on meta-analytic evidence revealing hypoactivation in the occipital cortex in PTSD, but not in other anxiety disorders (Etkin and Wager, 2007).

## Materials and methods

### Participants

We enrolled 125 patients with schizophrenia and 70 healthy controls with a negative family history for mental disorders at the National Psychiatry Center, Budapest, Hungary. The diagnosis was based on structured clinical interviews (First et al., 1996) and medical records. The patients with schizophrenia received antipsychotic medications at the time of testing (risperidone, olanzapine, quetipaine, haloperidol, aripriprazole, zuclopenthixol, amisulpride), which were converted to chlorpromazine-equivalent doses (Woods, 2003). The severity of the symptoms was characterized by the Brief Psychiatric Rating Scale (Overall and Gorham, 1962), and the Clinician-Administered PTSD Scale (CAPS) (Blake et al., 1990). We used the Four Factor Index of Social Status (Hollingshead, 1975). All participants had normal or corrected-to-normal visual acuity. From the 125 patients with schizophrenia, 21 patients (17%) exhibited comorbid PTSD (physical assault: *n* = 11; sexual assault: *n* = 7; sudden violent death of a spouse or friend: *n* = 1; traffic accident: *n* = 1; natural disaster: *n* = 1). In each patient PTSD was a current, and not a lifetime, comorbid condition. From the 21 SCZ + PTSD individuals, 7 patients (33%) also had major depressive disorder (MDD), and 4 patients (19%) had current substance misuse. From the 104 SCZ patients, 15 individuals (14%) had MDD, and 16 individuals (15%) had current substance misuse. The clinical and demographic data are summarized in Table 1. The study was done in accordance with the Declaration of Helsinki, and each participant gave written informed consent. The study was approved by the institutional ethics board.

**Table 1:** **Clinical and demographic characteristics**.

	**CONT (*n* = 70)**	**SCZ (*n* = 104)**	**SCZ + PTSD (*n* = 21)**
Male/female	45/25	60/44	9/12
Age (years)	38.6 (9.4)	40.1 (10.2)	39.6 (9.0)
Education (years)	12.3 (3.5)	11.0 (4.2)	11.6 (6.7)
Socioeconomic status	35.6 (8.9)	33.2 (9.3)	32.0 (10.5)
BPRS	–	42.4 (6.3)	45.2 (7.4)
Number of hospitalizations	–	4.9 (2.5)	5.1 (3.6)
Age at onset (years)	–	23.6 (5.2)	24.7 (6.1)
Chlorpromazine-equivalent antipsychotics (mg/day)	–	364.4 (192.4)	380.9 (173.1)
CAPS—Re-experiencing	–	–	17.9 (9.6)
CAPS—Avoidance	–	–	21.6 (8.7)
CAPS—Arousal	–	–	22.1 (8.4)

*Data are mean (standard deviation) with the exception of gender ratio. CONT, controls; SCZ, schizophrenia; PTSD, posttraumatic stress disorder; BPRS, brief psychiatric rating scale; CAPS, clinician-administered PTSD scale. The three groups did not differ in age, education, and socioeconomic status (p > 0.1, t-test). In the SCZ + PTSD group, females were overrepresented, although it was not significant compared with the other two groups (chi-square test, p > 0.05). SCZ and SCZ + PTSD patients did not differ in total BPRS scores (p > 0.1)*.

### Visual contrast sensitivity

**Figure 1:**
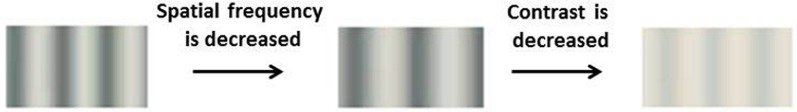
**Illustration of the principle of contrast sensitivity measurements.** The spatial frequency of a grating is reduced when the cycles (vertical bars with high and low luminance) under 1° is decreased. Contrast is reduced in a staircase procedure to the minimum level that can be detected by the participant. The inverse of this threshold is sensitivity. Temporal frequency is the contrast reversal of the grating (Hz).

The procedure, which is suitable for the reliable measurement of visual contrast sensitivity in individuals with less efficient general cognitive functions, has been described elsewhere (Kogan et al., 2004; Kéri and Benedek, 2009). We presented vertical sinusoidal luminance-contrast gratings on a gamma-corrected ViewSonic PF815 monitor. During visual contrast sensitivity measurements, the minimal luminance-contrast is measured, which is indispensable for the detection of the gratings (Figure 1). The first type of stimuli had low spatial frequency (SF) and high temporal frequency (TF) (0.3 cycle/degree and 18 Hz, respectively), and the second type of stimuli had high SF and low TF (10 cycles/degree and 1 Hz, respectively). The stimulus area was a circular window (mean luminance: 31 cd/m^2^, size: 8°). The initial Michelson-contrast was 12%. We used a Yes/No one-up/two-down staircase procedure (step size: 0.1 log contrast unit). The participants gave oral responses (grating is seen or not), and the experimenter entered all responses on the computer keyboard. We used catch trials to control for spurious responding. The staircase was finished when the slope and SD of the last 12 trials was less than the step size. The detection threshold was the mean of the last 12 reversals (for methodological details, see Kogan et al., 2004; Kéri and Benedek, 2009).

### Neuropsychological assessment

We used the Repeatable Battery for the Assessment of Neuropsychological Status (RBANS) battery, which is suitable for rapid and comprehensive evaluation of neurocognitive functions (Randolph, 1998; Gold et al., 1999; Juhász et al., 2003). The RBANS battery consists of 12 tests, combined to five index scores. Each index score is standardized (normal mean: 100, *SD* = 15 based on a normative study group of 200 healthy Hungarian volunteers, 20–80 years of age). The index domains are as follows: (1) immediate memory [word list learning (10 words repeated in four trials), story recall in two trials]; (2) language (confrontation naming of 10 pictures, category fluency); (3) visuospatial functions (figure copy, line orientation); (4) attention (digit span, digit-symbol coding); (5) delayed memory (delayed recall of the story, complex figure, and word list, recognition of the word list). The RBANS battery has two psychometrically matched forms; here we used version “A.”

### Data analysis

We used STATISTICA 11 (StatSoft, Inc., Tulsa) for data analysis. Data quality assumptions were evaluated with Kolmogorov–Smirnov test (normality of distribution) and Levene's tests (homogeneity of variance). Analyses of variance (ANOVAs) were used in the case of contrast sensitivity and RBANS data, followed by Tukey Honestly Significant Difference (HSD) tests, corrected for unequal sample sizes. We used two-tailed *t*-tests and chi-square tests for the comparison of demographic data. The relationship among the relevant variables was investigated using Pearson's product moment correlation coefficients (*r*). Where appropriate and informative, we also calculated Cohen's effect size values (*d*). The level of statistical significance was set at α < 0.05.

## Results

### Visual contrast sensitivity

**Figure 2:**
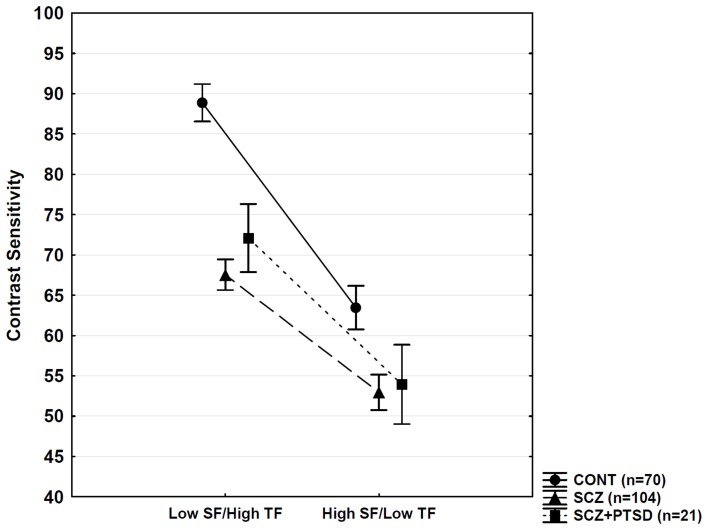
**Visual contrast sensitivity.** CONT, control; SCZ, schizophrenia; PTSD, posttraumatic stress disorder; SF, spatial frequency; TF, temporal frequency. Error bars indicate 95% confidence intervals. SCZ and SCZ + PTSD patients showed similar reductions in sensitivity relative to controls (*p* < 0.001, Tukey HSD for unequal Ns).

The ANOVA conducted on the contrast sensitivity data indicated significant main effects of group [*F*_(2, 192)_ = 95.47, *p* < 0.0001], stimulus type [*F*_(1, 192)_ = 208.83, *p* < 0.0001], and a Two-Way interaction between group and stimulus type [*F*_(2, 192)_ = 10.83, *p* < 0.001]. The *post-hoc* comparisons revealed that both SCZ and SCZ + PTSD groups exhibited reduced contrast sensitivity at low SF/high TF relative to healthy controls (*p* < 0.001), but there was no significant difference between the SCZ and SCZ + PTSD groups (*p* = 0.74). At high SF/low TF, the results were similar (CONT > SCZ = SCZ + PTSD; significant difference between CONT and SCZ groups: *p* < 0.001) (Figure 2). The Two-Way interaction between group and stimulus type appeared because the difference between patients (SCZ plus SCZ + PTSD) and controls was larger on the low SF/high TF condition (*d* = 1.47) than on the high SF/low TF condition (*d* = 0.83).

### Neuropsychological performance

**Figure 3:**
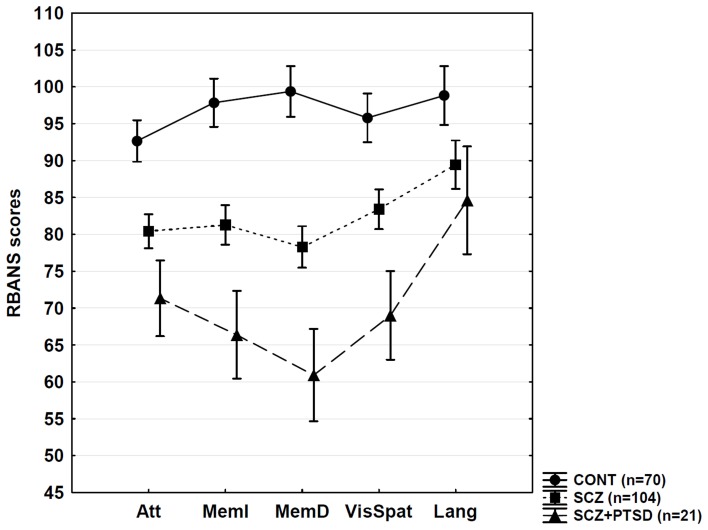
**Neuropsychological results.** CONT, control; SCZ, schizophrenia; PTSD, posttraumatic stress disorder; RBANS, Repeatable Battery for the Assessment of Neuropsychological Status; Att, attention; MemI, immediate memory; MemD, delayed memory; VisSpat, visuospatial; Lang, language. Error bars indicate 95% confidence intervals. For statistical details, see Table 2.

Figure 3 demonstrates the RBANS performance. The statistical analyses (One-Way ANOVAs followed by Tukey HSD for unequal Ns and Cohen's effect size values) are summarized in Table 2. Overall, SCZ patients exhibited significant deficits in attention, memory, visuospatial functions, and language. Patients with SCZ + PTSD showed more severe dysfunctions in attention, memory, and visuospatial functions relative to the SCZ group.

**Table 2:** **Neuropsychological results**.

**RBANS domain**	***F***	***df***	***p***	**Tukey HSD (unequal Ns) and Cohen's effect size (*d*)**
Attention	34.83	2.192	<0.0001	CONT > SCZ > SCZ + PTSD
				*d*_[CONT − SCZ]_ = 0.92
				*d*_[CONT − SCZ + PTSD]_ = 1.47
				*d*_[SCZ − SCZ + PTSD]_ = 0.75
Immediate memory	52.59	2.192	<0.0001	CONT > SCZ > SCZ + PTSD
				*d*_[CONT − SCZ]_ = 1.13
				*d*_[CONT − SCZ + PTSD]_ = 1.68
				*d*_[SCZ − SCZ + PTSD]_ = 1.0
Delayed memory	73.51	2.192	<0.0001	CONT > SCZ > SCZ + PTSD
				*d*_[CONT − SCZ]_ = 1.58
				*d*_[CONT − SCZ + PTSD]_ = 1.72
				*d*_[SCZ − SCZ + PTSD]_ = 0.70
Visuospatial functions	34.59	2.192	<0.0001	CONT > SCZ > SCZ + PTSD
				*d*_[CONT − SCZ]_ = 0.87
				*d*_[CONT − SCZ + PTSD]_ = 1.50
				*d*_[SCZ − SCZ + PTSD]_ = 0.93
Language	8.79	2.192	<0.001	CONT > SCZ = SCZ + PTSD
				*d*_[CONT − SCZ]_ = 0.53
				*d*_[CONT − SCZ + PTSD]_ = 0.88
				*d*_[SCZ − SCZ + PTSD]_ = 0.35

*The table present results from One-Way ANOVAs. RBANS, Repeatable Battery for the Assessment of Neuropsychological Status; CONT, healthy controls; SCZ, schizophrenia; PTSD, posttraumatic stress disorder. See also Figure 2*.

### Correlations and gender differences

Correlations among RBANS, contrast sensitivity, symptoms (BPRS and CAPS scores), and chlorpromazine-equivalent antipsychotic dose did not reach the level of statistical significance when SCZ and SCZ + PTSD patients were analyzed separately or together (−0.1 < *r* < 0.1, *p* > 0.1). There were no significant differences in any visual perceptual and neuropsychological measures between male and female patients (*p* > 0.1, *t*-tests).

### Effect of comorbid MDD and substance misuse

We compared SCZ and SCZ + PTSD patients with and without MDD/substance misuse on visual contrast sensitivity and RBANS performance. We did not find significant differences between patients with and without MDD/substance misuse in the SCZ and SCZ + PTSD groups (ANOVAs, *p* > 0.4).

## Discussion

The results of the present study indicate that SCZ + PTSD is associated with more severely disrupted memory, attention, and visuospatial functions compared with SCZ. In contrast, low-level visual processing is not affected by the presence of comorbid PTSD, which suggests that occipital hypoactivation observed in PTSD may not affect basic contrast processing (Etkin and Wager, 2007). In order to examine visual processing in more detail, patients with PTSD should be tested with the parallel application of psychophysical and neuroimaging techniques.

By the assessment of elderly individuals, Goodman et al. (2007) demonstrated that Holocaust survivor SCZ + PTSD patients scored lower on tests of speed of information processing, recognition memory, and general mental status than elderly SCZ patients without trauma exposure. Lysaker et al. (2001) found that individuals with schizophrenia-spectrum disorders with self-reported childhood sexual abuse performed worse in the case of working memory and executive functions than did individuals without self-reported abuse. Fan et al. (2008) also found a more severe generalized cognitive dysfunction in SCZ + PTSD than in SCZ, with a special reference to attention, working memory, and executive functions. However, two studies failed to find any evidence that SCZ + PTSD is associated with substantially increased neuropsychological impairment (Duke et al., 2010; Peleikis et al., 2012), although in the Duke et al. (2010) study SCZ + PTSD patients showed a qualitatively different neuropsychological profile compared with SCZ patients, which was influenced by differences in age, education, and symptoms. In the present study, we identified a relatively high number of SCZ + PTSD patients, which is consistent with the literature (Achim et al., 2011). SCZ + PTSD and SCZ patients displayed similar clinical and demographic characteristics, and, therefore, differences in neuropsychological performance cannot be attributed to these potential confounding variables. Comorbid MDD and substance misuse did not affect neuropsychological and visual performance. Correlation analysis also revealed negative results as regards symptoms and antipsychotic medications. A categorical comparison of symptom severity (mild, moderate, severe) is not possible because studies rarely use CAPS to characterize PTSD severity in schizophrenia.

Contrary to the differences in neuropsychological functions, SCZ + PTSD and SCZ patients demonstrated statistically indistinguishable visual contrast sensitivity deficits; in both groups, reduction in sensitivity was similar relative to controls, which was more pronounced at low SF/high TF. Several previous studies demonstrated altered visual contrast sensitivity in SCZ, but the data varied regarding the specific values measured at different SFs and TFs (e.g., Slaghuis, 1998; Kéri et al., 2002; Chen et al., 2003; Butler et al., 2009; Kiss et al., 2010; for a critical review, see Skottun and Skoyles, 2007, 2011). A more pronounced reduction in contrast sensitivity at low SF/high TF can be explained by the differential impairment of the magnocellular visual pathway, which project from the retina to the primary visual cortex via the lateral geniculate nucleus; this pathway participates in the processing of stimuli with low SF/high TF (Merigan and Maunsell, 1990). However, the magnocellular origin of contrast sensitivity alteration in SCZ has been debated (Skottun and Skoyles, 2007, 2011) because the magnocellular system shows a considerable overlap with other cells in the visual system regarding contrast and SF response. Nevertheless, evidence suggests that the cortical processing of low SFs is compromised in SCZ (Martínez et al., 2008; Kéri et al., 2012; Calderone et al., 2013), and that low SF achromatic gratings activate the putative magnocellular layers of the human lateral geniculate nucleus (Denison et al., 2012). The rapid development of high-resolution imaging techniques capable of identifying and visualizing the magnocellular layers may provide answer to this debate question.

The most important limitation of the present study is that no PTSD patients without SCZ were included. Therefore, it is not clear how PTSD itself affects neuropsychological and particularly visual functions in our study context. By conducting a direct comparison between SCZ + PTSD and PTSD alone, Duke et al. (2010) demonstrated much milder neuropsychological deficits in PTSD relative to PTSD + SCZ. Nevertheless, several studies have shown that PTSD itself is associated with compromised attention, memory, and executive functions (Golier and Yehuda, 2002), although the causal link between trauma and impaired cognition is controversial (Danckwerts and Leathem, 2003). Regarding low-level vision, even less information is available in PTSD. Hendler et al. (2003) showed that the visual cortex displayed more activation in PTSD when trauma-related images were exposed at below recognition threshold, which suggests that the earliest stage of visual information processing is corrupted in PTSD. However, it remains to be explored how PTSD patients react to emotionally neutral basic and more complex visual stimuli (e.g., Levy-Gigi and Kéri, 2012).

The second limitation was that we had to restrict the assessment of the symptoms, and we did not depict positive, negative, disorganized, and affective symptom separately. In general, however, BPRS and CAPS scores did not correlate with neuropsychological and visual contrast sensitivity performance. Finally, the sample size was still small in the SCZ + PTSD group, which may limit the generalizability of the findings, given that the symptoms, clinical course, and outcome of the illness show a high degree of individual variability and heterogeneity. Therefore, the results must be replicated and extended in a larger sample.

### Conflict of interest statement

The authors declare that the research was conducted in the absence of any commercial or financial relationships that could be construed as a potential conflict of interest.
